# A phase 1 evaluation of the pharmacokinetic/pharmacodynamic interaction of the anti-malarial agents KAF156 and piperaquine

**DOI:** 10.1186/s12936-017-2162-8

**Published:** 2018-01-05

**Authors:** F. Joel Leong, Jay Prakash Jain, Yiyan Feng, Budhaditya Goswami, Daniel S. Stein

**Affiliations:** 1 Novartis Institute for Tropical Diseases, Singapore, Singapore; 2 Novartis Healthcare Pvt Ltd, Hyderabad, India; 3 Novartis Institutes for BioMedical Research, Shanghai, People’s Republic of China; 4 Novartis Pharma, East Hanover, NJ USA; 5 Present Address: D3, Agency for Science, Technology and Research (A*STAR), Singapore, Singapore

**Keywords:** Malaria, QT interval, Piperaquine, KAF156

## Abstract

**Background:**

KAF156 is a novel imidazolopiperazine anti-malarial with activity against pre-erythrocytic liver stages, asexual and sexual blood stages. Based on in vitro data, a two-way pharmacokinetic interaction was hypothesized for KAF156 use in combination with piperaquine (PPQ) as both drugs are CYP3A4 substrates and inhibitors. Potential combination effects on the QT interval were also assessed.

**Methods:**

This was an open-label, parallel-group, single-dose study in healthy volunteers randomized to three parallel arms (1:1:1) of 800 mg KAF156 + 1280 mg PPQ, 800 mg KAF156 alone and 1280 mg PPQ alone. Triplicate ECGs were done up to 48 h post-dose. Routine safety and pharmacokinetic assessments were carried out up to 61 days.

**Results:**

Of the 72 healthy male subjects recruited, 68 completed the study. Co-administration of PPQ and KAF156 had no overall effect on AUC of either compound, but the Cmax values of both KAF156 (~ 23%) and piperaquine (~ 70%) increased. Both drugs given alone or in combination were well tolerated with no deaths or serious adverse events (SAEs). AEs were observed at the frequency of 87.5, 79.2 and 58.3% respectively for KAF156 + PPQ, PPQ and KAF156 arms. The most common AEs were nausea and headache. There were no Grade 3 or 4 events. There were no ECG related AEs, no QTcF interval > 480 ms and no QTcF interval increase from baseline > 60 ms. There was a positive ∆QTcF trend in the KAF156 + PPQ arm when either KAF156 or piperaquine concentration increases, but there was no significant difference between the combination arm and other arms in maximum ∆QTcF.

**Conclusions:**

No safety/cardiac risk or drug interaction was identified which would preclude use of a KAF156 and PPQ combination in future studies.

## Background

Despite a significant decrease in incidence over the last 5 years, malaria continues to have a significant health impact with 212 million cases worldwide in 2015, mostly in sub-Saharan Africa in children less than 5 years of age. In 2016, 91 countries were endemic for malaria, with approximately half within the World Health Organization (WHO) African region—sub-Saharan Africa experienced 90% of malaria cases and 92% of malaria deaths [1].

WHO guidelines recommend fixed dose combination therapy to decrease potential for development of resistance that can occur from either non-compliance or monotherapy. Drug resistance has occurred with all prior therapies developed for malaria and it has been detected to a limited extent for the artemisinin class, the current mainstay of treatment [2–7]. Despite emphasis on the use of combination therapies it is predicted that given the current incidence detection of artemisinin resistance in clinical settings [2], widespread artemisinin drug resistance will occur, as has been the historical experience for prior anti-malarials [8]. Therefore, development of new non-artemisinin based anti-malarial drugs is urgently needed.

KAF156 belongs to a novel class of anti-malarials (imidazolopiperazines) [9, 10]. Through a mechanism that remains to be determined, it kills both blood and liver schizonts, has both therapeutic and causal prophylactic activities in malaria mouse models, and blocks transmission to the Anopheles mosquito. It demonstrates nanomolar half maximal inhibitory concentration (IC50) values against all lab-adapted *Plasmodium falciparum* drug resistant strains as well as *Plasmodium vivax,* and *P. falciparum* clinical isolates (IC50 range = 5–15 nM) [11]. It has been dosed in healthy adult volunteers up to 600 mg daily for 3 days and 1200 mg as a single dose [12]. It has also demonstrated efficacy in uncomplicated adult malaria patients. With a multiple-dose regimen (400 mg daily for 3 days) median parasite clearance times (PCT) were 45 h (interquartile range, 42–48) in 10 patients with falciparum malaria and 24 h (interquartile range, 20–30) in 10 patients with vivax malaria. PCT was 49 h (interquartile range, 42–54) in 21 falciparum malaria patients after treatment with a single 800 mg dose [13].

KAF156 is absorbed rapidly with a Tmax of 1–4 h. It has over-proportional exposure in the dose range of 10–1200 mg. There is no significant impact of food on KAF156 exposure. Its mean elimination half-life is in range of 47.1–55.6 h. Pharmacokinetic properties are similar in both malaria patients and healthy subjects [12, 13].

Piperaquine (PPQ) is an approved anti-malarial drug as part of the combination product Eurartesim^®^ [14–17]. Piperaquine has long acting anti-malarial activity with relatively low levels of resistance, however, its major safety issue is a significant drug exposure-related increase in QT interval observed in trials that further increases when drug exposure is increased by food intake [18, 19].

KAF156 and piperaquine are both CYP3A4 inhibitors and primarily metabolized by CYP3A4. The inhibitory constant (Ki) values determined from the in vitro CYP3A4 inhibition study were 0.156–0.09 μM for KAF156 and piperaquine, respectively (Novartis data on file). However, SimCYP^®^ (Certara) modelling and simulation predicted no significant pharmacokinetic interaction for both KAF156 and piperaquine for in vivo combination use (i.e., < 1.25-fold change in AUC).

To evaluate piperaquine as a potential combination partner of KAF156 this study assessed the potential bidirectional interaction of KAF156 and piperaquine on each other’s pharmacokinetics in healthy subjects, and assessed the safety, including the cardiac safety (QTc prolongation), when KAF156 and piperaquine were given alone or in combination.

## Methods

### Study design

This was an open label, randomized, single dose, parallel-group and non-confirmatory study in healthy volunteers. It was conducted at Nucleus Network in Melbourne, Australia. The primary objective was to investigate the pharmacokinetic interaction potential between KAF156 and piperaquine in healthy subjects. Secondary objectives were to investigate the safety and tolerability of KAF156 and piperaquine alone and when co-administered in healthy subjects, and to investigate the potential effects on electrocardiogram (ECG) intervals (QT, PR, QRS) when KAF156 and piperaquine were given alone and in combination.

### Treatments and follow-up schedule

The proposed doses were 800–1280 mg for KAF156 and piperaquine, respectively. The KAF156 dose of 800 mg (KAF156 base equivalent) was given in the form of eight 100 mg strength tablets. Piperaquine was administered as tetraphosphate tetrahydrate. The piperaquine dose of 1280 mg (piperaquine tetraphosphate equivalent) was given in the form of four 320 mg strength tablets. The study consisted of a screening period of up to 26 days (Day − 28 to − 3), a baseline on Day -1, followed by a single dose treatment in 3 parallel treatment arms on Day 1, and a study completion evaluation. The total duration for each subject to complete the study including baseline without screening was approximately 61 days.

Given the known food effect for piperaquine (~ threefold increased exposure) and its QTc liability [20], all doses were given fasting. Subjects were admitted to the study site the night prior to dosing (approximately 12 h) in each arm for baseline evaluations. Eligible subjects fasted (i.e., no food or liquid except for water) for at least 10 h prior to administration of study drug on Day 1 and continued to fast for at least 4 h thereafter. No fluid intake apart from the fluid given at the time of drug intake was allowed from 2 h before until 2 h after dosing. Lunch and dinner was served approximately 4 and 8 h post dosing, respectively.

Subjects were domiciled at the site from baseline until 48 h post-dose. Subjects then returned to the site at the follow-up visits detailed in the assessment schedule, for up to a 61-day period, to undergo safety evaluations and PK sampling. Study completion evaluation was conducted after the last PK sampling on Day 11 for KAF156 or Day 61 for arms containing piperaquine.

### Subjects

The study population comprised healthy males, aged 18–45 years of age and in good health as determined by past medical history, physical examination, vital signs, ECG, and laboratory tests at screening. Subjects weighed at least 50 kg to participate in the study, with a body mass index (BMI) within the range of 18–30 kg/m^2^. The study was open to female subjects of non-childbearing potential, but none were recruited.

Exclusion criteria included use of other investigational drugs at the time of enrollment, or within five half-lives or within 30 days of enrollment; a history of clinically significant ECG abnormalities, screening/baseline Fridericia’s formula corrected QT interval (QTcF) elevation (> 430 ms for males, > 440 ms for females); women of child-bearing potential; smokers or smokeless tobacco users who were unwilling or unable to refrain from tobacco use during confinement to the clinical research centre and during required study visits/evaluations; haemoglobin levels below 12.0 g/dL at baseline; significant illness which did not resolve within 2 weeks prior to initial dosing; active disease; infections or conditions which may alter drug pharmacokinetics; renal or hepatic dysfunction; a history of drug or alcohol abuse within the 12 months prior to dosing, or clinical/laboratory evidence of such abuse.

A total of approximately 72 (n = 24/arm) subjects were planned to be enrolled and randomly assigned 1:1:1 into one of the three treatment arms:

- Arm I, 24 subjects received a single morning dose of 800 mg KAF156 + a single dose of 1280 mg piperaquine (KAF156 + PPQ).
- Arm II, 24 subjects received a single morning dose of 800 mg KAF156 (800 mg KAF156).
- Arm III, 24 subjects received a single morning dose of 1280 mg piperaquine (1280 mg PPQ).

### Safety assessment

Any potential relationship of drug exposure parameters to changes in ECG parameters was assessed. Safety assessments consisted of collecting all adverse events (AEs) and serious adverse events (SAEs), with their severity and relationship to study drug. They included regular monitoring of haematology, blood chemistry and urine performed at study centre and regular assessment of vital signs, physical condition, body weight and 12-lead electrocardiograms (ECG). Triplicate ECG assessments were performed at pre-dose and 2, 4, 8, 12, 24, and 48 h post-dose, while single ECGs were performed at the later time points (72 and 96 h and the end of study).

### Pharmacokinetic (PK) assessment

Plasma concentrations were determined at pre-dose and then at 0.5, 1, 2, 3, 4, 6, 8, 12, 24, 48, 72, 96, 144, 192, and 240 h for both KAF156 and piperaquine. Additional samples were taken at 336, 504, 672, 1008 and 1440 h post dose for piperaquine. Venous blood samples were collected either through an indwelling catheter or by venipuncture into K_2_EDTA-containing polyethylene tubes followed by gentle mixing and centrifugation between 2 and 8 °C for 10 min at approximately 1500 g. Tubes were stored on wet ice or cryoblock until centrifuged (within 60 min). Immediately after centrifugation, the supernatant plasma was transferred into 1.8 mL polypropylene screw-cap tubes which were placed in dry ice. The tubes were kept frozen at or below − 70 °C until bioanalysis. Plasma concentrations of KAF156 and piperaquine were determined by a validated liquid chromatography-tandem mass spectrometry (LC–MS/MS) method; the Lower Limit of Quantification (LLOQ) is 5 and 0.5 ng/mL for KAF156, and piperaquine, respectively.

The linearity ranges for KAF156 and PPQ are 1–5000 ng/mL, and 0.5–250 ng/mL, respectively. For the KAF156 assay, briefly, a 20.0 μL aliquot plasma sample was mixed with a 25.0 μL aliquot of the internal standard working solution [(M + 6)KAF156 500 ng/mL in 50% methanol]. A 200 μL aliquot of acetonitrile (ACN) was added to the mixture. Subsequently, the sample was centrifuged at 2000*g* for 10 min at 10 °C. A 150 μL aliquot of each supernatant was evaporated to dryness under nitrogen at 45 °C. The sample was reconstituted in a 300 μL aliquot of MeOH–water–formic acid (FA) (10:89.9:0.1, v/v/v). A 3.00 μL aliquot of the sample was injected onto the LC–MS/MS system. The piperaquine method has already been described elsewhere [21].

The accuracy and precision for both KAF156 and piperaquine were within acceptable limits for study validation. For calibration standards, both KAF156 (5.00, 10.0, 50.0, 250, 500, 1500, 4000 and 5000 ng/mL) and piperaquine (0.500, 1.00, 2.50, 10.0, 50.0, 100, 225 and 250 ng/mL) had bias within the acceptable range of ± 20.0% at the LLOQ and ± 15.0% at the other concentration levels. Similarly, the 3 levels of quality control samples for both KAF156 and piperaquine had bias within the acceptable range of ± 15% for at least 2/3 of the individual values.

The following PK parameters were determined using the actual recorded sampling times and non-compartmental method(s) with Phoenix WinNonlin (Version 6.4): Cmax, Tmax, AUClast, AUCinf, T1/2, Vz/F and CL/F for KAF156 and piperaquine from the plasma concentration–time data.

The linear trapezoidal rule was used for AUC calculation. Regression analysis of the terminal plasma elimination phase for the determination of T1/2 included at least three data points after Cmax. If the adjusted R^2^ value of the regression analysis of the terminal phase was to be less than 0.75, no values were to be reported for T1/2, AUCinf and CL/F. If extrapolated AUCinf was more than 20% for KAF156 or more than 40% for piperaquine (due to its long terminal half-life) AUCinf and related parameters were not included in statistical analysis.

PK/PD relationships, using the individual concentrations and QTcF changes from baseline at each time point, were explored to establish the relationship between QTcF and drug exposure.

### Statistical methods

Primary PK parameters were Cmax and AUClast. AUCinf was also estimated and reported; however, it was not considered as a primary PK parameter. The log-transformed primary PK parameters of KAF156 and piperaquine were analysed using a linear fixed effect model with treatment as the fixed effect. The analysis was performed using the natural log scale for PK parameters and the difference in adjusted means along with 90% confidence interval compared for the following:

- KAF156 800 mg with piperaquine 1280 mg *vs.* KAF156 800 mg.
- KAF156 800 mg with piperaquine 1280 mg *vs.* piperaquine 1280 mg.

All results for the defined contrasts were back-transformed to the original scale to present adjusted geometric mean ratios and the corresponding 90% confidence intervals.

### Secondary variables

#### Analysis of ECG parameters over time points

A key secondary endpoint for this trial was change from baseline in QTcF. Bazett’s formula corrected QT interval (QTcB) analysis was limited to descriptive statistics and reported only as a summary table. The baseline was calculated from the pre-dose triplicate ECGs. At pre-dose and 2, 4, 8, 12, 24, and 48 h post-dose triplicate ECG assessments were collected. The mean of triplicate QTcF values was calculated and used for all subsequent calculations and statistical evaluations. The endpoint for this analysis was calculated by subtracting the mean of triplicated QTcF pre-dose from post-dose assessment for each subject at each time point.

For 72 and 96 h post-dose time points, only a single assessment was performed. This endpoint was obtained by subtracting the mean of triplicate pre-dose QTcF value from the post-dose QTcF value for each subject and time point.

The change from baseline for QTcF for each time point was analysed using a linear model with treatment as the fixed effect and baseline (baseline was taken as 0 h on Day 1) as covariate in the model separately. The difference in adjusted means along with the 90% two-sided CI was calculated for KAF156 800 mg + piperaquine 1280 mg vs. KAF156 800 mg, and KAF156 800 mg + piperaquine 1280 mg vs. piperaquine 1280 mg.

An arithmetic mean (± standard deviation (SD)) plot for change from baseline QT data (in Y axis) over several observed time points (in X axis) was performed for each treatment.

#### Analysis of maximal change in ECG parameters

A similar analysis was performed for maximal change from baseline for QTcF separately for KAF156 concentrations alone and in the presence of piperaquine, and for piperaquine concentrations alone and in the presence of KAF156, using a linear model with treatment as the fixed effect and concentration of KAF156 and piperaquine at maximal change as the covariate. The difference in adjusted means along with the 90% CI was calculated for KAF156 800 mg + piperaquine 1280 mg vs. KAF156 800 mg, and KAF156 800 mg + piperaquine 1280 mg vs. piperaquine 1280 mg. For the endpoint maximal change from baseline the same model was explored using AUCinf or Cmax as covariate in the model instead of plasma concentration.

## Results

### Subject demographics

Seventy-two subjects were enrolled, 68 subjects (94.4%) completed the study and 4 subjects (5.6%) discontinued (2 subjects each in the KAF156 + PPQ arm and 1280 mg PPQ arm). The primary reason for discontinuation was “subject/guardian decision”. All subjects were male with a mean age of approximately 26 years old (range 19–45 years), and weight range 56.4–105.7 kg. 70.8% were Caucasian, 4.2% Black, 15.3% Asian, and 9.7% were other unspecified ethnicities. There were no major differences in demographic characteristics among treatment arms.

### Safety and tolerability

There were no serious adverse events (SAEs) or deaths. The KAF156 + PPQ cohort had the highest incidence of adverse events (21/24, 87.5%), mostly attributable to nausea. This was followed by the PPQ cohort (19/24, 79.2%), then the KAF156 cohort (14/24, 58.3%). In the KAF156 + PPQ cohort the most common AEs were nausea (41.7%), upper respiratory tract infection (25.0%), and headache (16.7%). Headache was the most common AE in the KAF156 cohort (20.8%) and PPQ cohorts (33.3%) (Table 1).

**Table 1:** Incidence of adverse events by preferred term

Preferred term	800 mg KAF156 + 1280 mg PPQ	800 mg KAF156	1280 mg PPQ	Total
	N = 24	N = 24	N = 24	N = 72
	n (%)	n (%)	n (%)	n (%)
Number of subjects with at least one AE	21 (87.5)	14 (58.3)	19 (79.2)	54 (75.0)
Headache	4 (16.7)	5 (20.8)	8 (33.3)	17 (23.6)
Nausea	10 (41.7)	3 (12.5)	2 (8.3)	15 (20.8)
Upper respiratory tract infection	6 (25.0)	2 (8.3)	7 (29.2)	15 (20.8)
Abdominal pain	3 (12.5)	1 (4.2)	0	4 (5.6)
Dizziness	2 (8.3)	0	2 (8.3)	4 (5.6)
Diarrhoea	1 (4.2)	1 (4.2)	1 (4.2)	3 (4.2)
Dyspepsia	1 (4.2)	1 (4.2)	1 (4.2)	3 (4.2)
Somnolence	0	1 (4.2)	2 (8.3)	3 (4.2)
Fatigue	1 (4.2)	0	1 (4.2)	2 (2.8)
Pyrexia	0	0	2 (8.3)	2 (2.8)
Visual impairment	1 (4.2)	1 (4.2)	0	2 (2.8)
Vomiting	1 (4.2)	0	1 (4.2)	2 (2.8)
Abdominal discomfort	1 (4.2)	0	0	1 (1.4)
Alanine aminotransferase increased	0	1 (4.2)	0	1 (1.4)
Catheter site erythema	0	1 (4.2)	0	1 (1.4)
Conjunctival haemorrhage	0	1 (4.2)	0	1 (1.4)
Cough	0	1 (4.2)	0	1 (1.4)
Dry skin	0	0	1 (4.2)	1 (1.4)
Dyskinesia	0	0	1 (4.2)	1 (1.4)
Foreign body	1 (4.2)	0	0	1 (1.4)
Gastroenteritis	0	1 (4.2)	0	1 (1.4)
Gastroesophageal reflux disease^a^	0	0	1 (4.2)	1 (1.4)
Infected bite	1 (4.2)	0	0	1 (1.4)
Insomnia	0	0	1 (4.2)	1 (1.4)
Ligament sprain	1 (4.2)	0	0	1 (1.4)
Lymphadenopathy	1 (4.2)	0	0	1 (1.4)
Muscle spasms	0	0	1 (4.2)	1 (1.4)
Myalgia	0	0	1 (4.2)	1 (1.4)
Neck pain	1 (4.2)	0	0	1 (1.4)
Oropharyngeal pain	0	0	1 (4.2)	1 (1.4)
Phlebitis	0	1 (4.2)	0	1 (1.4)
Rash erythematous	1 (4.2)	0	0	1 (1.4)
Sleep disorder	0	0	1 (4.2)	1 (1.4)
Toothache	0	0	1 (4.2)	1 (1.4)
Viral infection	0	0	1 (4.2)	1 (1.4)

A subject with multiple adverse events (AEs) is counted only once in the “at least one AE” row

A subject with multiple AEs with the same preferred term is counted only once for that preferred term & treatment

Preferred terms are sorted in descending frequency

^a^Reflux oesophagitis began before dosing but was exacerbated following dosing and labelled ‘drug-related’

Nausea was disproportionally higher in the KAF156 + PPQ arm (41.7%) than the 800 mg KAF156 (12.5%) or 1280 mg PPQ (8.3%) arms. Vomiting was present in a single subject in the KAF156 + PPQ and 1280 mg PPQ arms only (4.2%), with vomiting in the KAF156 + PPQ arm occurring minutes after dosing. Most subjects with AEs had Grade 1 events. A total of 16 subjects had at least one Grade 2 AE. There were no Grade 3 or 4 events.

Laboratory abnormalities were generally not of clinical significance. Hypereosinophilia up to 2.33 × ULN was seen distributed across all dosing arms in up to 23 subjects with no apparent difference among arms. This study was conducted during hay fever season in Melbourne and none of the cases were considered clinically-significant. Elevated creatine kinase levels (ranging from Grade 1 to Grade 3 abnormalities), occurred in most of the affected subjects before study drug administration, was not associated with AEs, was generally transitory, and occurred across dosing arms with no obvious pattern or dose relationship. There was an AE of Grade 1 elevated AST, suspected to be drug-related occurring in one subject in the 800 mg KAF156 arm. All his ALT readings, including those at screening and baseline were also Grade 1 elevations. Vital signs showed no significant changes in mean/median values over time which were within normal ranges, and no notable differences among the treatment arms.

### ECG results

#### Individual results

There were no ECG related AEs, no QTcF interval > 480 ms, and no QTcF interval increase from baseline > 60 ms. There was no difference in heart rate among the treatment arms. Mean maximal change from baseline (Table 2): Maximum increase in QTcF from baseline was observed at 24 h post-dose for both KAF156 + PPQ and KAF156 monotherapy arm; the mean increases from pre-dose (Day 1—0 h) was 7.9 and 6.6 ms, respectively. For the PPQ monotherapy arm, the maximum increase from pre-dose was found to be 1.8 ms (mean) at 4 h post-dose. The maximum QTcF change, irrespective of time point, was highest in the KAF156 + PPQ arm (median 13.7 ms, maximum 26 ms) followed by the 800 mg KAF156 arm (median 8.0 ms, maximum 44 ms) then the 1280 mg PPQ (median 7.0 ms, maximum 23.7 ms). The KAF156 + PPQ arm also had the highest mean change (12.5 ms) compared with the single treatment arms (800 mg KAF156:10.8 ms; 1280 mg PPQ:8.2 ms).

**Table 2:** Summary statistics of maximal change from pre-dose in QTcF (safety analysis set)

Treatment	Statistics	QTcF change from pre-dose (msec)
KAF156 800 mg + PPQ 1280 mg	N	24
	Mean (SD)	12.5 (7.84)
	CV%	62.51
	Median	13.7
	(Min, max)	(− 8.7, 26.00)
KAF156 800 mg	N	24
	Mean (SD)	10.8 (9.83)
	CV%	91.18
	Median	8.0
	(Min, max)	(− 1.7, 44.00)
PPQ 1280 mg	N	24
	Mean (SD)	8.2 (8.29)
	CV%	101.63
	Median	7.0
	(Min, max)	(− 7.3, 23.67)

QTcF = Maximum change from post treatment in QTcF

**Fig. 1:**
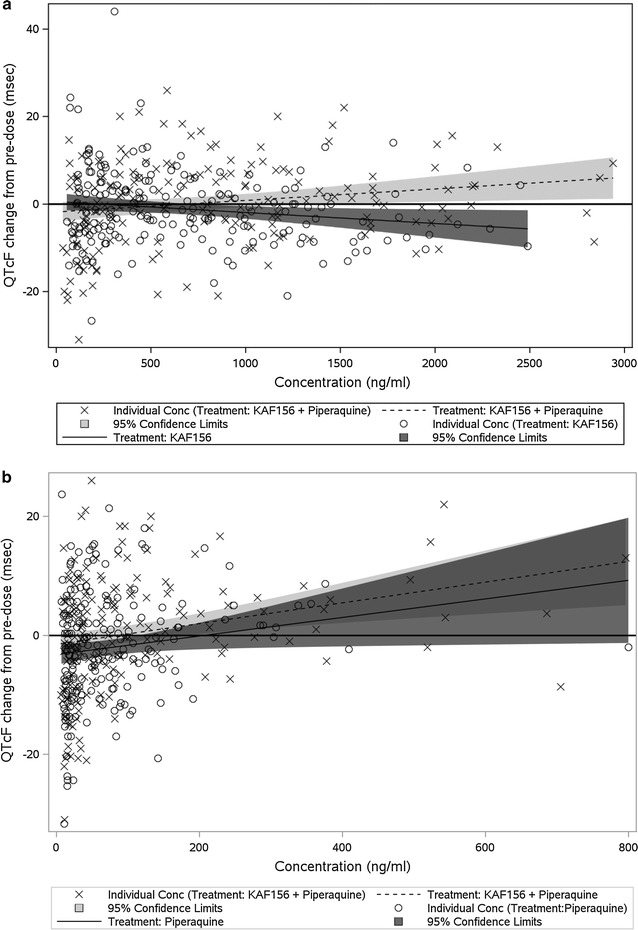
Individual drug concentration and its relationship with QTcF change from baseline when given as monotherapy and as combination; **a** for KAF156, **b** for PPQ. Open circles represent the monotherapy and cross marks represent the combination treatment. The dark gray and light gray shaded areas represent the 95% confidence interval for monotherapy and combination respectively

Concentration effect relationship for QTcF: To understand the concentration effect relationship, individual concentrations vs. ∆QTcF on specific time points and maximum QTcF, change from pre-dose (∆QTcF) vs. Cmax were plotted. Additionally, an ANCOVA model was fitted over pre-dose corrected QTcF values, with Cmax (of KAF156 then piperaquine) as covariate and respective treatments as fixed effects. There was a positive ∆QTcF trend in the KAF156 + PPQ arm when either KAF156 or piperaquine concentration increases, but there was no significant difference between the combination arm and other arms in maximum ∆QTcF (p > 0.05). In Fig. 1 the potential concentration-effect relationships for QTcF change from baseline is explored as individual KAF156 (panel A) or piperaquine (panel B) concentrations vs. maximal change from baseline in QTcF. In both analyses the slope of the relationship is not significantly different for the monotherapy vs. combination comparison. Therefore, no evidence of a synergistic effect on QTc interval when KAF156 and piperaquine are combined was found.

### Pharmacokinetic assessment

**Fig. 2:**
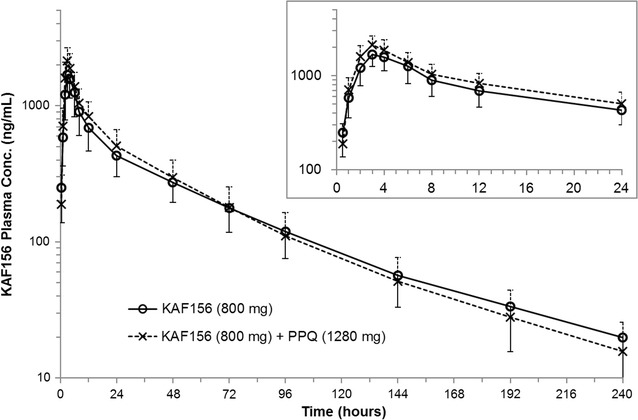
Arithmetic mean (SD) plasma concentration time profiles for KAF156 according to time and treatment group. Inset, first 24 h after dosing. Solid lines with open circles represent the KAF156 concentrations when given as monotherapy and dashed lines with cross marks represent KAF156 concentrations when given in combination with PPQ

**Fig. 3:**
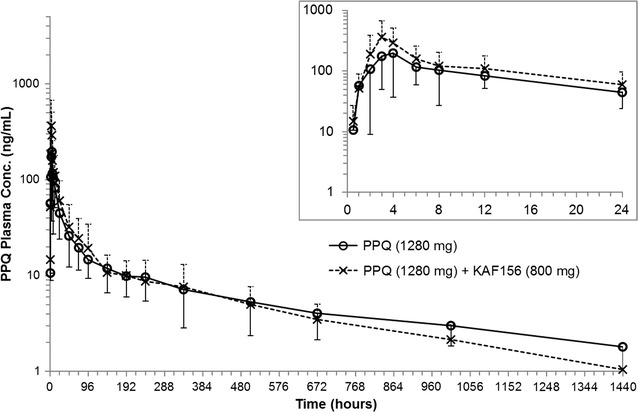
Arithmetic mean (SD) plasma concentration time profiles for piperaquine according to time and treatment group. Inset, first 24 h after dosing. Solid lines with open circles represent the PPQ concentrations when given as monotherapy and dashed lines with cross marks represent PPQ concentrations when given in combination with KAF156

Figures 2, 3 show the exposure vs. time profiles for KAF156 and piperaquine when administered as either a monotherapy or part of the combination therapy. The corresponding PK parameters are shown in Tables 3 and 4. There was no noteworthy alteration in the extent of exposure (AUC) of KAF156 and piperaquine when given in combination compared to their administration as single agent. KAF156 Cmax was 1.23-fold (90% CI 1.10, 1.37) higher in the combination arm compared to KAF156 alone arm. Piperaquine Cmax was 1.69 (90% CI 1.16, 2.45) fold higher in the combination arm compared to monotherapy arm however it should be noted that piperaquine Cmax was highly variable (CV% ~ 70%). There was no increase in the T1/2 of either drug when given in combination compared to their single drug arms; however, there was shortening of either median Tmax or range of Tmax for both the drugs in combination compared to their respective single drug arms.

**Table 3:** PK parameters for KAF156 in the presence and absence of piperaquine

PK parameter^a^ (unit)	800 mg KAF156 + 1280 mg PPQ N = 23 (mean, SD, CV%)	800 mg KAF156 N = 24 (mean, SD, CV%)
Cmax (ng/mL)	2270 ± 496 (21.8%) [n = 23]	1850 ± 401 (21.7%) [n = 24]
AUClast (h µg/mL)	48.2 ± 14.4 (30.0%) [n = 22]	44.4 ± 12.7 (28.7%) [n = 24]
AUCinf (h µg/mL)	50.1 ± 15.0 (29.9%) [n = 21]	46.2 ± 13.9 (30.0%) [n = 24]
AUC0-24 h (h µg/mL)	22.8 ± 5.41 (23.7%) [n = 22]	19.1 ± 4.86 (25.4%) [n = 24]
AUC0-72 h (h µg/mL)	38.2 ± 10.2 (26.6%) [n = 22]	33.0 ± 8.67 (26.2%) [n = 24]
AUC0-168 h (h µg/mL)	47.0 ± 13.7 (29.0%) [n = 21]	42.2 ± 11.8 (27.9%) [n = 24]
Tmax (h)	3.00 (2.00–4.02) [n = 23]	3.00 (2.00–6.00) [n = 24]
T1/2 (h)	52.4 ± 11.1 (21.2%) [n = 21]	57.9 ± 10.5 (18.2%) [n = 24]
CL/F (mL/h)	17,500 ± 5760 (32.9%) [n = 21]	18,700 ± 5210 (27.8%) [n = 24]
Vz/F (L)	1360 ± 689 (50.7%) [n = 21]	1530 ± 417 (27.2%) [n = 24]

AUC0-t h, The area under the plasma concentration–time curve from time zero to time ‘t’ where t is a defined time point after administration

AUCinf, The area under the plasma concentration–time curve from time zero to infinity

AUClast, The area under the plasma concentration–time curve from time zero to the time of the last quantifiable concentration

Cmax, The observed maximum plasma concentration following drug administration

CL/F, The apparent systemic (or total body) clearance from plasma following extravascular administration

T1/2, The terminal elimination half-life

Tmax, The time to reach the maximum concentration after drug administration

Vz/F, The apparent volume of distribution during the terminal elimination phase following extravascular administration

^a^All PK parameter values are presented as mean ± SD (CV %) [n] except Tmax which is presented as median (range) [n]. n is number of subjects providing reliable estimate of the parameter

**Table 4:** PK parameters for piperaquine in the presence and absence of KAF156

PK parameter^a^ (unit)	800 mg KAF156 + 1280 mg PPQ N = 23 (mean, SD, CV%)	1280 mg PPQ N = 24 (mean, SD, CV%)
Cmax (ng/mL)	409 ± 299 (73.2%) [n = 23]	233 ± 169 (72.3%) [n = 24]
AUClast (h µg/mL)	10.8 ± 6.14 (56.8%) [n = 22]	10.3 ± 4.16 (40.2%) [n = 23]
AUCinf (h µg/mL)	11.9 ± 5.25 (44.1%) [n = 19]	12.0 ± 5.31 (44.2%) [n = 17]
AUC0-24 h (h µg/mL)	2.94 ± 1.81 (61.5%) [n = 22]	2.09 ± 0.993 (47.4%) [n = 24]
AUC0-72 h (h µg/mL)	4.73 ± 2.89 (61.0%) [n = 22]	3.49 ± 1.56 (44.8%) [n = 24]
AUC0-168 h (h µg/mL)	6.35 ± 3.67 (57.9%) [n = 21]	4.82 ± 1.95 (40.4%) [n = 23]
Tmax (h)	3.00 (2.00–4.05) [n = 23]	4.00 (3.00–8.00) [n = 24]
T1/2 (h)	469 ± 170 (36.2%) [n = 19]	509 ± 142 (27.9%) [n = 17]
CL/F (mL/h)	127,000 ± 53,100 (41.7%) [n = 19]	126,000 ± 52,700 (41.9%) [n = 17]
Vz/F (L)	82,200 ± 34,500 (42.0%) [n = 19]	89,100 ± 32,800 (36.8%) [n = 17]

Abbreviations as per Table 3

^a^All PK parameter values are presented as mean ± SD (CV%) [n] except Tmax which is presented as median (range) [n]. n is number of subjects providing reliable estimate of the parameter

## Discussion

This study examined the safety, potential pharmacokinetic and QTcF interactions of KAF156 and piperaquine when dosed alone or in combination. There were no clinically relevant AE’s found for either agent when dosed either alone or in combination (Table 1) and the safety profiles resembled past studies performed with KAF156 [12, 13] or piperaquine given as a single dose in healthy volunteers [21].

One of the major safety concerns for the use of these agents in combination is the potential for QT prolongation given the known risk for piperaquine and the potential risk from in vitro screens of KAF156 (e.g., hERG inhibition). For this reason, the study incorporated triplicate ECG analyses and planned to evaluate ECG changes as a function of exposure response relationships. There was no evidence of a synergistic effect when the agents were given together: piperaquine but not KAF156 had a positive slope for exposure vs. increase in QTcF.

**Fig. 4:**
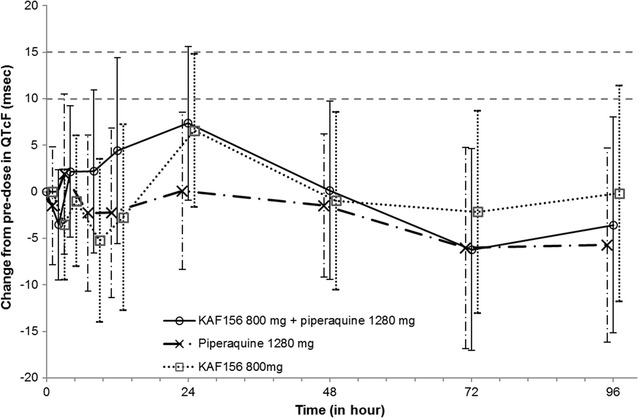
Arithmetic mean (SD) QTcF change time profile from pre-dose profile by treatment. Combination is indicated by the solid line with circles, KAF156 monotherapy by the dotted line with squares, and piperaquine monotherapy by the dash-dot line with cross marks

In a previous study examining the safety, PK, and PD relationship for KAE609 and piperaquine [21], the mean maximal change from baseline for piperaquine in the regression model was 7.47 ms, which is similar to the 8.49 ms observed in this trial. The overall change from baseline vs time for QTcF (Fig. 4) for piperaquine is less consistent than in the earlier study and the difference between the piperaquine and combination arms was greater in that study. It is unclear why these differences between studies were found as the study site, use of triplicate ECGs and analysis are the same. The main difference is that given the longer half-life of KAF156 compared with KAE609, triplicate ECGs were performed for a longer time interval after dosing in this trial.

The PK profiles of each agent are consistent with the prior data for each agent [12, 21] when dosed under fasting conditions in healthy volunteers. There were statistically significant increases in the Cmax values of KAF156, 1.23-fold (90% CI 1.10, 1.37), and piperaquine, 1.69-fold (90% CI 1.16, 2.45), when dosed in combination. Given the lack of a relationship of the increased Cmax values of each agent to QTcF increase, as noted in the earlier analysis, it is unlikely that there is clinical relevance to the Cmax increase observed for either drug.

Overall, no safety/cardiac risk or drug–drug interaction was identified which would preclude use of a KAF156 and PPQ combination in future studies. As both KAF156 and piperaquine have demonstrated efficacy in prior clinical trials, the combination could be explored in clinical trials without dose adjustment or concern for increased cardiac arrhythmia risk as a non-cross resistant alternative to artemisinin combination therapy in the treatment of blood-stage falciparum malaria.
